# Understanding Interface Dipoles at an Electron Transport Material/Electrode Modifier for Organic Electronics

**DOI:** 10.1021/acsami.1c13172

**Published:** 2021-09-23

**Authors:** Yongzhen Chen, Xianjie Liu, Slawomir Braun, Mats Fahlman

**Affiliations:** Laboratory of Organic Electronics, Department of Science and Technology, Linköping University, 60174 Norrköping, Sweden

**Keywords:** interface dipole, electron transport material, hydrogen bond, molecular orientation, organic electronics

## Abstract

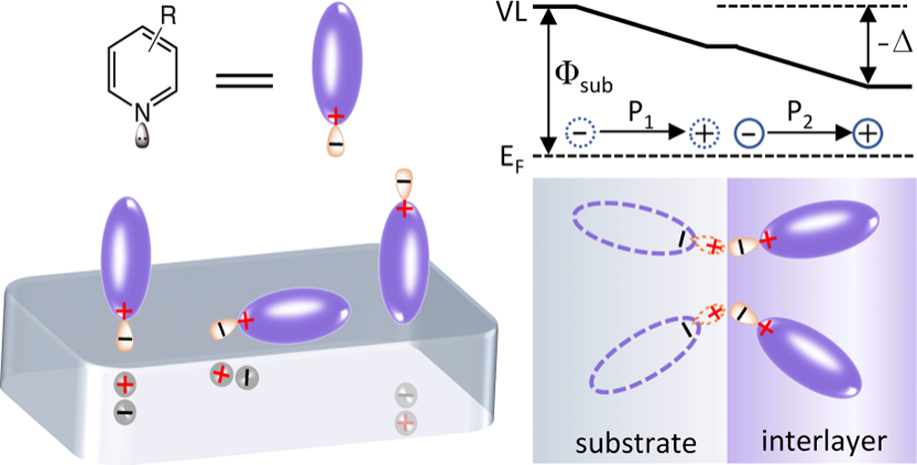

Interface dipoles formed at an electrolyte/electrode interface have been widely studied and interpreted using the “double dipole step” model, where the dipole vector is determined by the size and/or range of motion of the charged ions. Some electron transport materials (ETMs) with lone pairs of electrons on heteroatoms exhibit a similar interfacial behavior. However, the origin of the dipoles in such materials has not yet been explored in great depth. Herein, we systematically investigate the influence of the lone pair of electrons on the interface dipole through three pyridine derivatives B2–B4PyMPM. Experiments show that different positions of nitrogen atoms in the three materials give rise to different hydrogen bonds and molecular orientations, thereby affecting the areal density and direction of the lone pair of electrons. The interface dipoles of the three materials predicted by the “double dipole step” model are in good agreement with the ultraviolet photoelectron spectroscopy results both in spin-coated and vacuum-deposited films. These findings help to better understand the ETMs/electrode interfacial behaviors and provide new guidelines for the molecular design of the interlayer.

## Introduction

Interfacial engineering plays a crucial role in developing efficient and stable organic-based (opto-)electronic devices such as light-emitting diodes and photovoltaic cells.^1−3^ In a multilayer stacked device, the organic/electrode interfaces mainly control the injection or extraction of the charge carriers. Due to mismatch between the lowest unoccupied molecular orbital of organic semiconductors (OSCs) and the work function of stable cathodes, and the generally inferior electron transport properties of OSCs, the organic/cathode interface attracts significant attention.^4,5^ Incorporating a dipole layer at the electrode interface is a common strategy to achieve Ohmic contacts and minimize the potential barrier. Inorganic salts, such as LiF, CaF_2_, and Cs_2_CO_3_, have been extensively used as a dipole layer and show excellent electron-injection properties.^6−8^ However, considering the high diffusivity of these salts that result in exciton annihilation and poor stability and their limited applications in inverted and flexible devices, several promising organic compounds have been introduced as interlayers that exhibit excellent performance comparable to the inorganic salts, such as small-molecule electron transport materials (ETMs) bathophenanthroline (BPhen), bathocuproine (BCP), polymers polyethyleneimine, poly[(9,9-bis(3′-(*N*,*N*-dimethylamino)propyl)-2,7-fluorene)-*alt*-2,7-(9,9-dioctyl-fluorene)], and some of their polyelectrolyte derivatives.^9−15^

After modification with such materials, an interface dipole will form and decrease the work function of the cathode electrode. Some of these interfaces have been investigated in detail and described using the “double dipole step” model, that is, two electric dipoles with the same normal vector created by the separated positively and negatively charged species on the interlayer side and their image charges on the electrode side.^16−19^ The direction of the dipole depends on the size and/or range of motion of the two types of charged species. The mechanism is easy to understand for electrolytes, where both the free and covalently bonded ions can act as the charged species. For polymers and small-molecule ETMs without charged ions, however, the charged species consist of heteroatoms with a lone pair of electrons.^17,18^ Although all the surface analytical results are in good agreement with the double dipole step model, there is still a lack of detailed results on the origin of the dipole. It can be reasonably inferred from the model that the magnitude of the interface dipole is dependent on the distance and areal density of the positively and negatively charged species in the vertical direction at equilibrium. In this context, one can adjust the dipole through altering the molecular orientation and/or the number of lone pairs in the molecule.

The pyridine derivatives BPyMPM are a series of commonly used ETMs in organic light-emitting diodes and solar cells.^20,21^ Each molecule contains four pyridine substituents, in which N atoms with high electronegativity feature an in-plane lone pair of electrons on the sp^2^ orbital. By comparing with the molecule BPhen,^18^ we believe that BPyMPM should produce a similar interface dipole in contact with the electrode. In addition, for the vacuum-deposited films, it has been demonstrated that the molecular planes of B3PyMPM and B4PyMPM are oriented nearly parallel to the substrate surface due to the formation of intermolecular C–H···N hydrogen bonds in the films that facilitate molecular stacking. However, it is difficult to form intermolecular hydrogen bonds in the B2PyMPM film because the nitrogen atoms in the pyridine rings are located inside of the molecule, and the molecular orientations in the film are random.^21,22^ Accordingly, the areal density and direction of the lone pairs in the films are different between B2PyMPM and B3–B4PyMPM and the magnitude of the dipoles hence should also be different. In this work, we explore the intermolecular hydrogen bonding and molecular orientation of the B2PyMPM and B3–B4PyMPM films and their effect on interface double-dipole formation. Combined with the reported theoretical simulations and comparison between spin-coated and vacuum-deposited films, the BPyMPM ETMs are systematically investigated.

## Experimental Details

### Materials and Sample Preparation

Bis-4,6-(3,5-dipyridylphenyl)-2-methylpyrimidine (BPyMPM) derivatives are purchased from Lumtec. Corp. with purity higher than 99% and used as received. The conducting substrates used in the study span a wide range of work function from aluminum (Φ = 3.8 eV) to UVO-treated gold (Φ = 5.7 eV). The ZnO and PEDOT:PSS substrates are achieved by spin-coating the corresponding solutions onto the indium tin oxide (ITO)-covered glass and annealing at 125 °C. Quartz and CaF_2_ substrates are used for the UV–vis and Fourier-transform infrared spectroscopy (FTIR) measurements, respectively. Before the deposition of films, all substrates are cleaned in ultrasonic baths of deionized water, acetone, and isopropanol for 15 min, respectively. The spin-coated films are made from chloroform solution in the atmosphere with a concentration of 5.0 mg mL^–1^ for B2–B3PyMPM and 2.5 mg mL^–1^ for B4PyMPM. The thickness of all films is maintained around 10–15 nm through changing the rotation speed. The vacuum-deposited films are fabricated in a vacuum sublimation chamber with a pressure below 5 × 10^–7^ mbar, and the deposition rate is around 0.02–0.04 nm s^–1^. After preparation, the films are taken out for photoelectron spectroscopy or other characterizations.

### Thin Film Characterization

The film thickness is measured using a Dektak surface profilometer. UV–vis absorption spectra are measured with PerkinElmer Lambda 900. The FTIR spectra are measured with a Bruker Equinox 55 spectrometer in transmission mode. Ultraviolet photoelectron spectroscopy (UPS) experiments are performed on a home-designed spectrometer using monochromatized He I radiation with an excitation energy of 21.22 eV. X-ray photoelectron spectroscopy (XPS) is carried out using the Scienta-200 hemispherical analyzer using monochromatized Al Kα radiation of 1486.6 eV energy. All photoelectron spectroscopy measurements were carried out with a base pressure lower than 1 × 10^–9^ mbar. Near edge X-ray adsorption fine structure spectroscopy (NEXAFS) is carried out at the FlexPES beamline of the 1.5 GeV storage ring in the MAX IV laboratory, Sweden. The spectra are collected in partial electron mode with a multichanneltron plate detector with different retard voltages for selected elements.

## Results and Discussion

**Figure 1:**
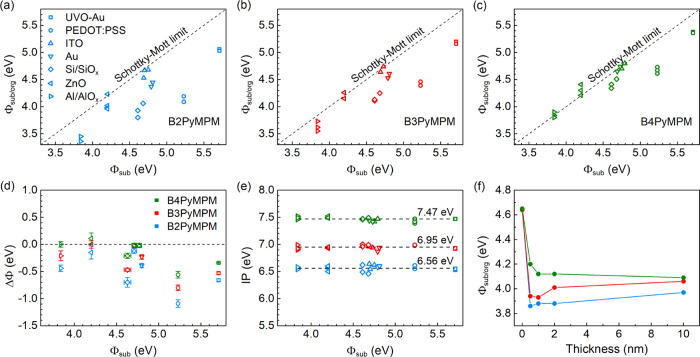
(a–c) Dependence of work function (Φ_sub/org_) of spin-coated films on the substrate with different work functions (Φ_sub_): (a) B2PyMPM; (b) B3PyMPM; and (c) B4PyMPM. Comparison of (d) interface dipoles (ΔΦ) and (e) ionization potentials between B2–B4PyMPM, ΔΦ = Φ_sub/org_ – Φ_sub_. (f) Evolution of work function upon increasing the thickness of BPyMPM films vacuum-deposited on Si/SiO_*x*_.

First, we study the modification of work function induced by these three materials. Figure 1a–c displays the dependence of the work function of BPyMPM (Φ_sub/org_) spin-coated onto various conducting substrates (Φ_sub_). All results are derived from the secondary electron cutoff of the UPS spectra. Unlike BCP and BPhen,^18,23^ these three materials show negative dipoles of different sizes, depending on the type of substrate applied. The ultrawide bandgap of the materials covers the work function of the substrates used (see Figure S1), so the UPS results obtained here are located in the vacuum level alignment regime and any change in work function is not caused by integer charge transfer.^24,25^

The interface dipoles (ΔΦ) derived from the change of work function are summarized in Figure 1d. We find that the interface dipoles of the three materials exhibit an identical variation tendency on all substrates: ΔΦ_B2_ > ΔΦ_B3_ > ΔΦ_B4_. In detail, B2PyMPM shows the largest interface dipole on all substrates, which means the work function of the substrate will be minimized by B2PyMPM. In particular, the interface dipole reaches −0.9 eV when it is deposited on PEDOT:PSS. B3 and B4PyMPM have smaller interface dipoles compared to B2PyMPM. Especially the latter, the work function remains fairly unchanged after depositing B4PyMPM on Al/AlO_*x*_, ZnO, ITO, and Au. When comparing spin-coated films on different substrates, we find that the decrease in work function is negligible when the three materials are deposited on ITO and ZnO. Therefore, these materials are unsuitable for use as a cathode interlayer in inverted devices with ITO or ITO/ZnO as the bottom electrode. Surprisingly, when the films are prepared using the thermal sublimation method, the interfacial behavior is rather different. As shown in Figure S2, the interface dipoles produced by the three materials are roughly the same on a specific substrate. They all show the smallest interface dipoles on ZnO and the largest interface dipoles on Si/SiO_*x*_, PEDOT:PSS, and UVO-Au, which are also observed in the spin-coating results.

The ionization potentials (IPs) derived from the UPS spectra are presented in Figure 1e. Each material has a quite stable value and the respective IP is almost independent of the substrate. The average IPs of the spin-coated B2–B4PyMPM films are 6.56, 6.95, and 7.47 eV, respectively, wherein the first two are in accordance with the sublimation results (see Figure S3). However, the B4PyMPM films prepared using the sublimation method have a lower IP of 7.32 eV, which is also consistent with the previous report.^20^ The optical band gaps derived from the UV–vis absorption spectrum of the two types of B4PyMPM films are identical, as shown in Figure S4, indicating the electron affinity changes along with IP. B2 and B3PyMPM are included for comparison, which also show the same absorption between two preparation methods.

To elucidate the formation of the interface dipole, the thickness dependence of work function is studied by UPS characterization. Here, we only measure the sublimated films because it is difficult to prepare the ultrathin films, for example, monolayer and bilayer, using the spin-coating method. The preparation method likely changes the morphology, resulting in different sizes of the interface dipole, but the nature of the dipole should be inherent and independent of the deposition method. The work functions derived from the UPS spectra of the three materials deposited on Si/SiO_*x*_ step-by-step are shown in Figure 1f (for more details, see Figure S5). As expected, we observe that the work functions all drop sharply after depositing 0.5 nm-thick films and remain roughly constant with the further increase of thickness with a slight fluctuation in B3PyMPM when the thickness increases to 2.0 nm. This result reveals that the interface dipoles are mainly located in the first monolayer of the films, in accord with the double dipole step model. The position of the N atoms in the pyridine rings, which is the only difference between the three materials, causes the change of the interface dipole. The formation of the double dipole step is attributed to the N atoms with lone pairs of electrons close to the substrate surface. One dipole moment formed by the N nuclei and the lone pairs points from the substrate surface to the organic film and the other one formed by their image charges shows the same direction.^16,18^ The N atoms far away from the substrate contribute little to the interface dipole.

**Figure 2:**
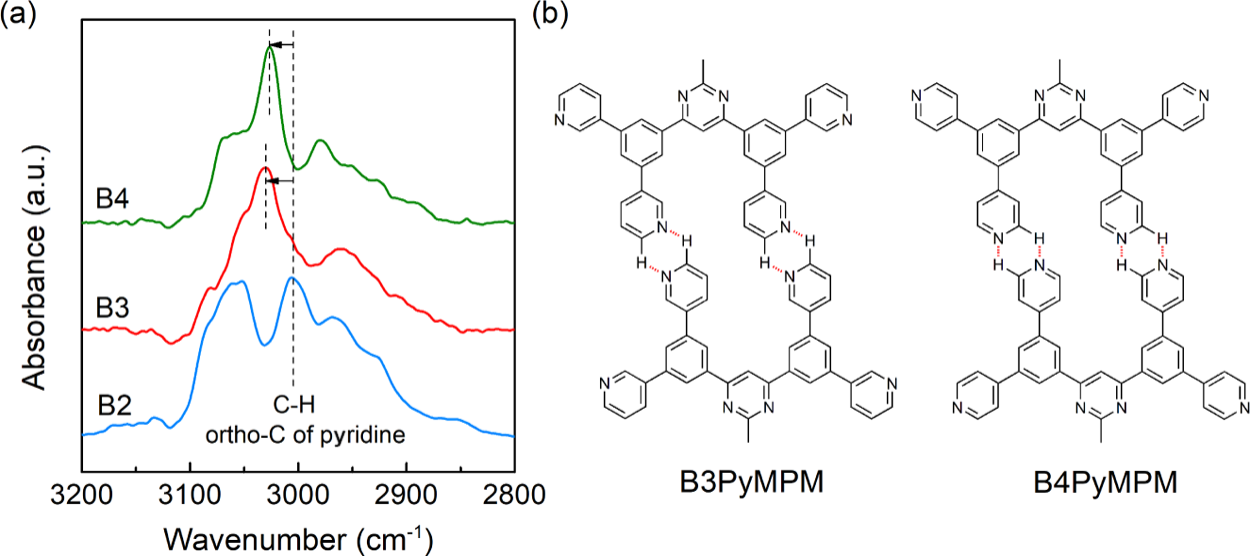
(a) FTIR spectra of the spin-coated films of B2–B4PyMPM. The blue shift of the main bands in B3 and B4PyMPM is marked by arrows. (b) Examples of intermolecular hydrogen bonds formed in B3 and B4PyMPM films.

According to the model, the magnitude of the dipole is dependent on the areal density and direction of the lone pairs on the substrate surface. Any intra- or intermolecular interactions involving the lone pair of electrons and affecting the molecular orientation should be taken into account. To figure out the difference in the dipole formation of the three materials, FTIR measurements are carried out, first on the spin-coated films. Instead of directly looking at the bonding of N atoms, we focus on the absorption of C–H vibrations. As shown in Figure 2, the spectra around 3000 cm^–1^ arising from the aromatic C–H stretching mode are significantly different for the three materials. According to the theoretical predictions,^26,27^ the blue shift of the main peaks in B3 and B4PyMPM is attributed to the formation of intermolecular hydrogen bonds (C–H···N). The same results are also observed in the vacuum-deposited films as shown in Figure S6 as also reported by Yokoyama et al.^21^ From the density-functional theory simulations in that report, the main peak is assigned to the C–H vibrational mode at the ortho-position of the pyridine rings, as shown in Figure 2b. The weak hydrogen bond has been intensively studied through other similar oligopyridine derivatives and the experimental results are in good agreement with the theoretical calculations.^22,28,29^ The formation of hydrogen bonds occupies a part of lone pairs of electrons, reducing the number of charged species capable of generating a double dipole step. Accordingly, the slight increase in the work function of B3PyMPM at 2.0 nm observed in Figure 1f can be interpreted by the increase in hydrogen bonds in the first layer with the deposition of the later molecules. This is also consistent with the more ratios of C=N carbons in a 1.2 nm-thick film derived from XPS spectra compared to the 15 nm-thick film and spin-coated film, as shown in Figure S7. There are fewer hydrogen bonds in the B2PyMPM film due to the greater steric hindrance when the N atoms are located inside of the molecule. Therefore, the interface dipoles of B3 and B4PyMPM should be smaller than that of B2PyMPM, in conformity to the UPS results of the spin-coated films.

**Figure 3:**
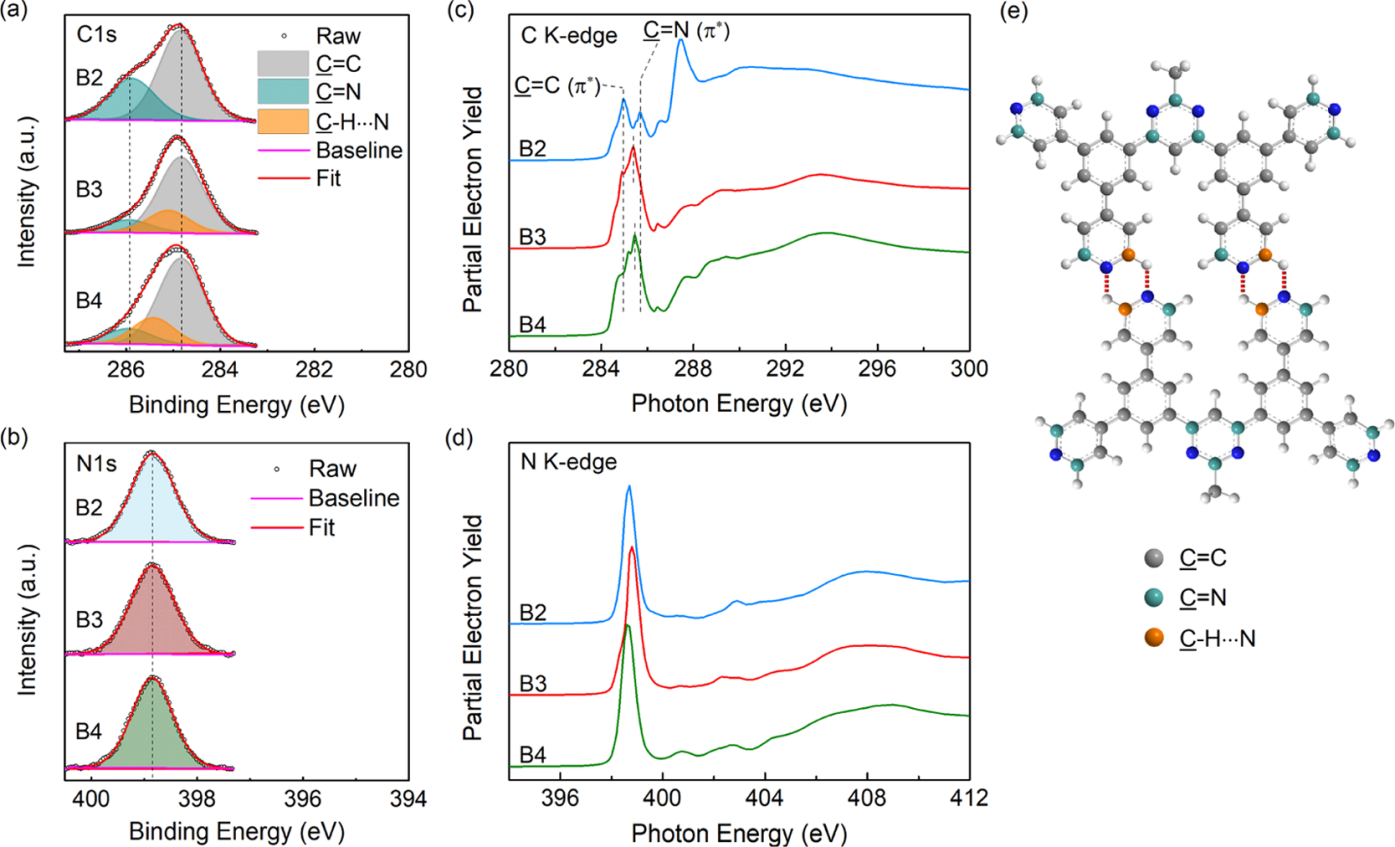
(a) C 1s and (b) N 1s XPS spectra of spin-coated films on ITO. The spectra of B3 and B4PyMPM are aligned to that of B2PyMPM. (c) C K-edge and (d) N K-edge NEXAFS spectra of spin-coated films. (e) Three types of carbon in the H-bonded B4PyMPM dimer correspond to C with different core levels.

To further confirm the formation of a hydrogen bond in these films, the core level of carbon and nitrogen is investigated by XPS measurement. It has been demonstrated that typical H-bonding interactions affect the local electronic state of the hydrogen bond donor and acceptor atoms, for example, N, O, F, and so forth.^30−32^ The C 1s spectra of the spin-coated films with a simple curve fitting are shown in Figure 3a. We can easily find that the C 1s spectrum of B2PyMPM is composed of two peaks, one with a higher intensity at 284.87 eV and the other with a lower intensity at 285.93 eV (see Table 1). Although there are a dozen types of carbon atoms theoretically in a molecule,^33^ the two peaks are roughly assigned to the C atoms that are not directly attached (C=C) and attached (C=N) to the N atom, corresponding to the atoms marked in green and blue, respectively, in Figure 3e. This is because the ortho C atoms are susceptible to the electronegative N atom and become electron deficient. The area ratio of the two peaks is 2.33:1, fitting neatly into the stoichiometric ratio (26 C=C carbon atoms and 11 C=N carbon atoms in one molecule). The C 1s spectra of B3 and B4PyMPM are aligned to the B2PyMPM spectrum, where we still observe the C=C and C=N features at the same binding energy (BE) (see Table 1). However, the relative intensity of the C=N peak is reduced, and a new feature appears between the original two peaks. From the proportion of the new feature, we can infer that it is produced by a part of the C=N carbon atoms shifting to lower BE (see the atoms marked in yellow in Figure 3e). The shift of ∼0.5–0.7 eV is reasonable for weak hydrogen bonding C–H···N and in good agreement with previous reports.^34^Figure 3b shows the aligned N 1s spectra of the three materials. Their symmetric curves reveal that no new nitrogen is produced by hydrogen bonding in B3 and B4PyMPM, manifesting that hydrogen bonding has little effect on the electronic charge of the acceptor atoms, which has also been observed in some other materials.^34−36^

**Table 1:** C 1s Peak Fitting Parameters of the Spin-Coated Films Including BE, Area Ratio, and Full Width at Half-Maximum (fwhm)

	states	BE (eV)	fwhm (eV)	ratio (%)
B2PyMPM	C=C	284.87	1.10	69.9
	C=N	285.93	1.10	30.1
B3PyMPM	C=C	284.83	1.10	69.9
	C=N	285.93	1.10	11.2
	C–H···N	285.08	1.04	18.9
B4PyMPM	C=C	284.86	1.13	69.4
	C=N	285.98	1.12	11.8
	C–H···N	285.45	1.04	18.8

The difference in core levels of the three materials is verified by the NEXAFS spectroscopy, which provides fingerprints for identifying chemical structures. Figure 3c,d shows the carbon and nitrogen K-edge NEXAFS spectra of the spin-coated films on Si/SiO_*x*_. From the C K-edge spectrum of B2PyMPM, two prominent splitting peaks are observed at 285.0 and 285.7 eV, stemming from the unsaturated C 1s → π* transitions. According to the theoretical study of the NEXAFS of pyridine and pyrimidine,^37−39^ the lower energy peak is attributed to the C=C carbon atoms and the higher energy peak is attributed to the C=N carbon atoms (see Figure 3e), which are in line with the XPS fitting results. We can also find two dominant peaks in the same region of B3 and B4PyMPM, due to the higher resolution of the NEXAFS as compared to the C 1s XPS spectra. However, they are much closer than the case of B2PyMPM. When the left peaks from C=C 1s → π* transitions are aligned, the right ones shift by 0.30 and 0.25 eV toward lower energy for B3 and B4PyMPM, respectively. Like XPS, this shift is caused by a part of H-bonded C=N carbons with increased electron density as the donor of hydrogen bonds. The N 1s NEXAFS spectra of the three materials exhibit the characteristic features of pyridine and pyrimidine with a dominant peak at ca. 398.8 eV, arising from the N 1s → π* transition. We also perform the XPS and NEXAFS measurements for the vacuum-deposited films (Figure S8 and Table S1), which show the same results as the spin-coated ones.

The blue shift of the C–H vibrational frequency and the decreased BE of the C=N carbon core level firmly demonstrate the presence of hydrogen bonds in B3 and B4PyMPM films, which results in the smaller interface dipoles. However, we cannot yet compare the dipoles between B3 and B4PyMPM through the H-bonding effect. It has been reported that the weak hydrogen bonds in planar molecules not only restrict the molecular freedom thereby achieving a more planar structure, but also facilitate the formation of continuous H-bonding networks with special orientations.^28,40−42^ The optical anisotropies of B3 and B4PyMPM films suggest they prefer to be face-on oriented in the vacuum-deposited film.^21^ Due to the in-plane property of the sp^2^ orbital, the direction of the lone pairs is determined by the orientation of the pyridine rings. Therefore, the measurement of molecular arrangement will help to further analyze the interface dipoles of the three materials.

**Figure 4:**
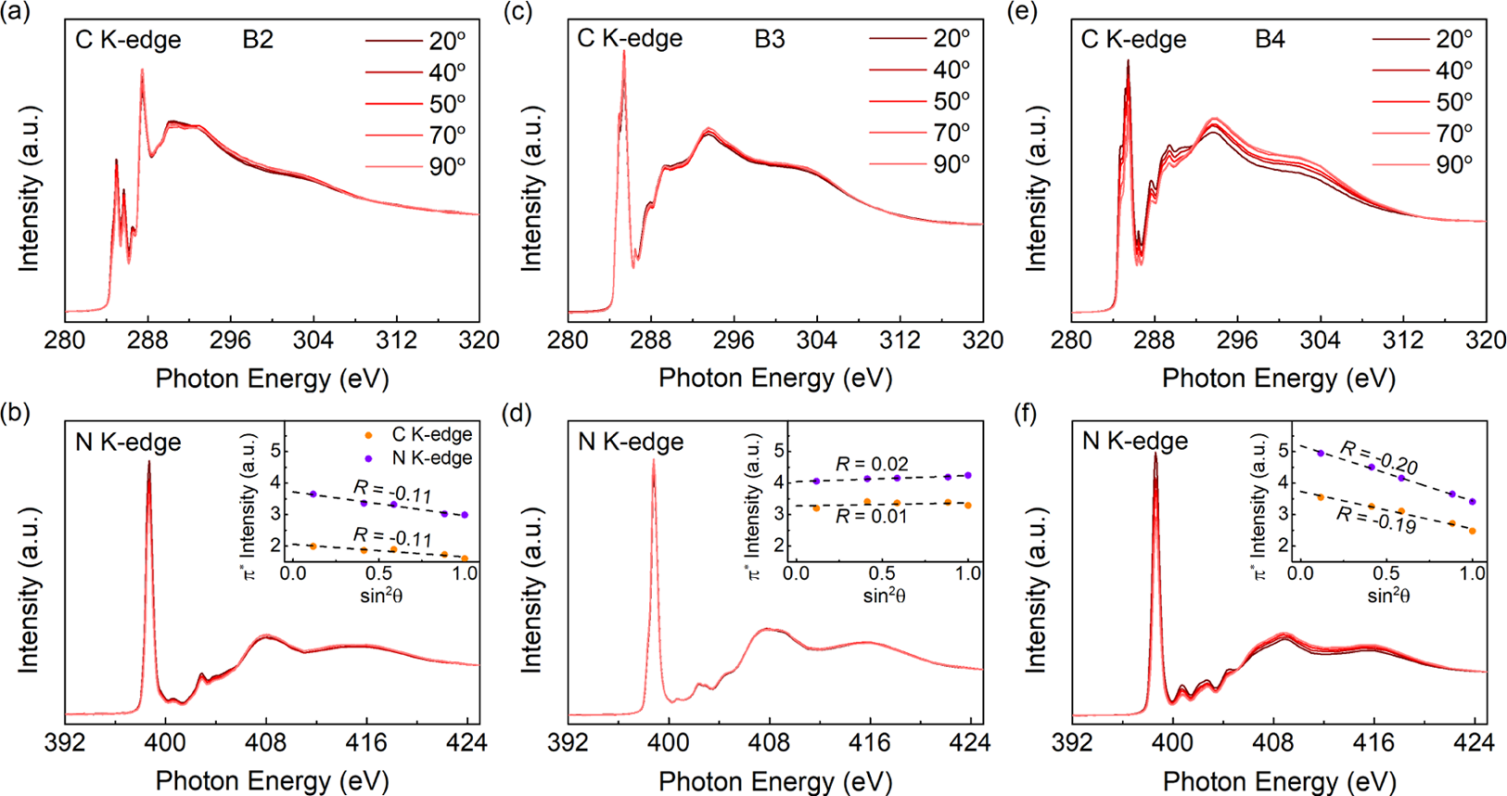
Angle-resolved NEXAFS spectra of carbon (a,c,e) and nitrogen (b,d,f) by varying incident angles from 20 to 90° measured from vacuum-deposited films. The insets show the dichroic ratio R calculated from the fitting results. Note that the thickness of all films is around 10–15 nm.

NEXAFS spectroscopy provides an effective way to study the molecular orientation through the final state of the X-ray excitation, such as π* and σ*.^43^ The π* resonance intensity (I) is proportional to the squared dot product of the incident electrical field vector and the transition dipole moment (TDM). For a given linearly p-polarized light and molecule, the intensity depends only on the incident angle (θ) and the tilt angle (α), where θ refers to the angle between the surface normal and the electric field vector of the light and α is the angle of the TDM relative to the surface normal. To quantify the molecular orientation, a dichroic ratio expressed by *R* = (*I*_90°_ – *I*_0°_)/(*I*_90°_ + *I*_0°_) is introduced, where *I*_90°_ and *I*_0°_ are the intensities at the incident angles of 90 and 0°, respectively.^44^ Due to the TDM of the π* orbital is oriented perpendicular to the conjugated plane, *R* varies from −1 for a fully face-on orientation to 0.7 for a fully edge-on orientation. *R* = 0 represents the magic angle (α = 54.7°) or completely random orientation. The NEXAFS spectra measured from θ = 20 to 90° and the calculated *R* values are presented in Figure 4. Three materials exhibit rather different molecular orientations in the films. The molecular plane of B2PyMPM shows an insignificant face-on orientation with *R* = −0.11 derived both from the C and N NEXAFS spectra, corresponding to a tilt angle of α = 52.2°. Because it is implausible to prepare a highly ordered film using the spin-coating method to form a magic angle arrangement, *R* = 0.01 for B3PyMPM indicates the film is completely random. In contrast, the π* intensity of B4PyMPM decreases significantly with increasing incident angle, showing a preferential face-on orientation to the substrate with *R* = −0.26 and α = 49.9°. The more horizontal molecular plane reduces the dipole moment formed by the nitrogen nucleus and lone pair in the normal direction of the substrate. As a result, the interface dipole of B4PyMPM is decreased further compared to that of B3PyMPM.

This result is different from the reported optical anisotropies of the vacuum-deposited films, which display both B3 and B4PyMPM are preferential face-on oriented derived from the anisotropy of the transition dipole moments and molecular polarizabilities.^21^ Here, we also investigate the orientations of the vacuum-deposited thin films with a thickness of 1.2 nm and the results are shown in Figure S9. Compared to the spin-coated films, B2PyMPM converts to a more edge-on orientation (i.e., increased tilt angle of the molecular plane from the substrate), while both B3 and B4PyMPM exhibit clear face-on orientation. Schematic diagrams for the molecular orientation of all films are given in Figure S10. The similar H-bonding effect and face-on orientation of B3 and B4PyMPM result in the similar interface dipoles in the vacuum-deposited films. Because the N atoms are located inside the B2PyMPM molecule, the lone pairs of electrons are unable to move close to the substrate in an edge-on molecule, as shown in Figure S11. Conversely, the most stable conformation formed by the pyridine ring with a certain rotation angle allows the lone pair to contact the substrate. As a result, the interface dipole of B2PyMPM is reduced to the same level as that of B3 and B4PyMPM in the vacuum-deposited films. This is also the main reason that the interface dipole of B2PyMPM is much smaller than those of BPhen (−1.4 eV) and BCP (−1.6 eV),^18,23^ even though the former is less affected by the hydrogen bonding.

## Conclusions

To figure out the origin of the dipole formed at the ETM/electrode interface, we investigate the influence of the number and orientation of the lone pair of electrons through a series of pyridine derivatives, BPyMPM. The interface dipoles measured by UPS show a uniform trend for the films spin-coated on various conducting substrates: ΔΦ_B2_ > ΔΦ_B3_ > ΔΦ_B4_, whereas the vacuum-deposited films show similar interface dipoles for all three materials. Through the C–H stretching vibration and carbon core-level analysis, we confirm the presence of weak hydrogen bonds C–H···N both in the spin-coated and vacuum-deposited B3 and B4PyMPM films, which will occupy a part of lone pairs of electrons. From the angle-resolved NEXAFS spectra, the preferential face-on orientation makes the interface dipole of B4PyMPM smaller than that of B3PyMPM in the spin-coated films. B2PyMPM has the largest areal density of lone electron pairs and optimal orientation, enabling it to achieve the largest interface dipole. However, in the vacuum-deposited films, the edge-on orientation of B2PyMPM prevents the lone electron pairs to move closer to the substrate, and B3PyMPM has the same face-on orientation as B4PyMPM, resulting in the similar interface dipoles for all of them. These results indicate that the interface dipole formed by ETMs can be accurately explained using the “double dipole step” model, that is, the nuclei and lone electron pairs of the heteroatoms contribute to the interface dipole.

## Supporting Information Available

Chemical structures; interfacial behaviors of vacuum-deposited films; comparison of the UV–vis spectra between spin-coated and vacuum-deposited films; thickness dependence of UPS spectra, FTIR, XPS, and NEXAFS spectra of the vacuum-deposited films; C 1s peak-fitting parameters; schematics of the molecular orientations; and contact of B2PyMPM molecules with substrates (PDF)
