# Supplementary material for: Understanding Interface Dipoles at an Electron Transport Material/Electrode Modifier for Organic Electronics
Source: ACS Appl Mater Interfaces. 2021 Sep 23;13(39):47218–25. doi: 10.1021/acsami.1c13172 (PMC8498986; doi:10.1021/acsami.1c13172)
Supplement: Supplementary file 1 — am1c13172_si_001.pdf [file am1c13172_si_001.pdf]

Supporting information

Understanding interface dipoles at electron transport material/electrode modifier for organic electronics

Yongzhen Chen, Xianjie Liu, Slawomir Braun, Mats Fahlman*

Laboratory of Organic Electronics, Department of Science and Technology, Linköping University, 60174 Norrköping, Sweden.

*Corresponding Author: yongzhen.chen@liu.se

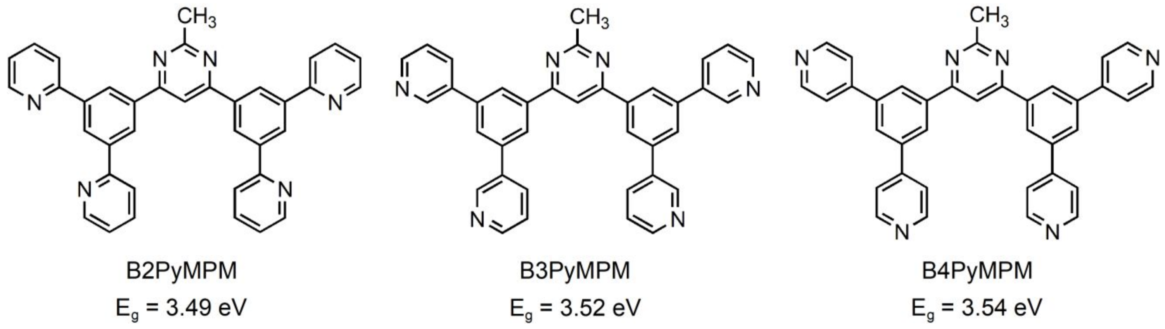

Figure S1. Chemical structures of 4,6-Bis(3,5-di(pyridin-2-yl)phenyl)-2-methylpyrimidine (B2PyMPM), 4,6-Bis(3,5-di(pyridin-3-yl)phenyl)-2-methylpyrimidine (B3PyMPM) and 4,6-Bis(3,5-di(pyridin-4-yl)phenyl)-2-methylpyrimidine (B4PyMPM). The optical band gap (E_g) is derived from the cutoff of the UV–vis absorption.

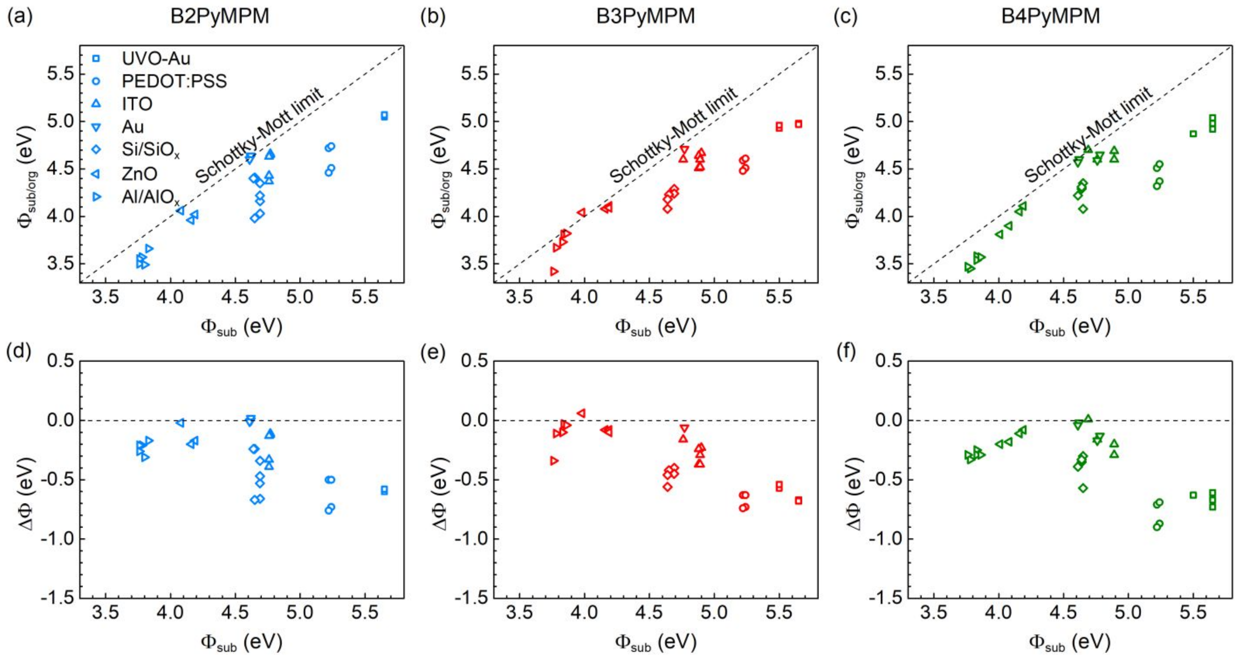

Figure S2. Dependence of (a–c) work function ($\Phi_{sub/org}$) and (d–f) interface dipole ($\Delta\Phi$) of vacuum-deposited films on the substrate with different work functions (Φ_{sub}): (a,d) B2PyMPM; (b,e) B3PyMPM; (c,f) B4PyMPM.

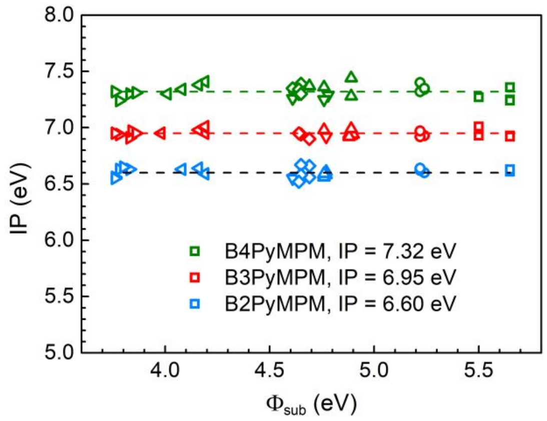

Figure S3. Summary of ionization potential (IP) of the vacuum-deposited films on different substrates.

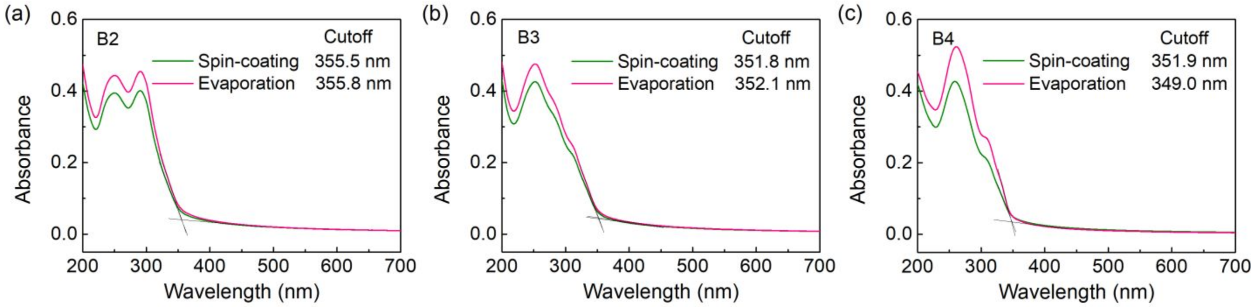

Figure S4. Comparison of the UV−vis spectra between spin-coated and vacuum-deposited films for (a) B2PyMPM, (b) B3PyMPM and (c) B4PyMPM.

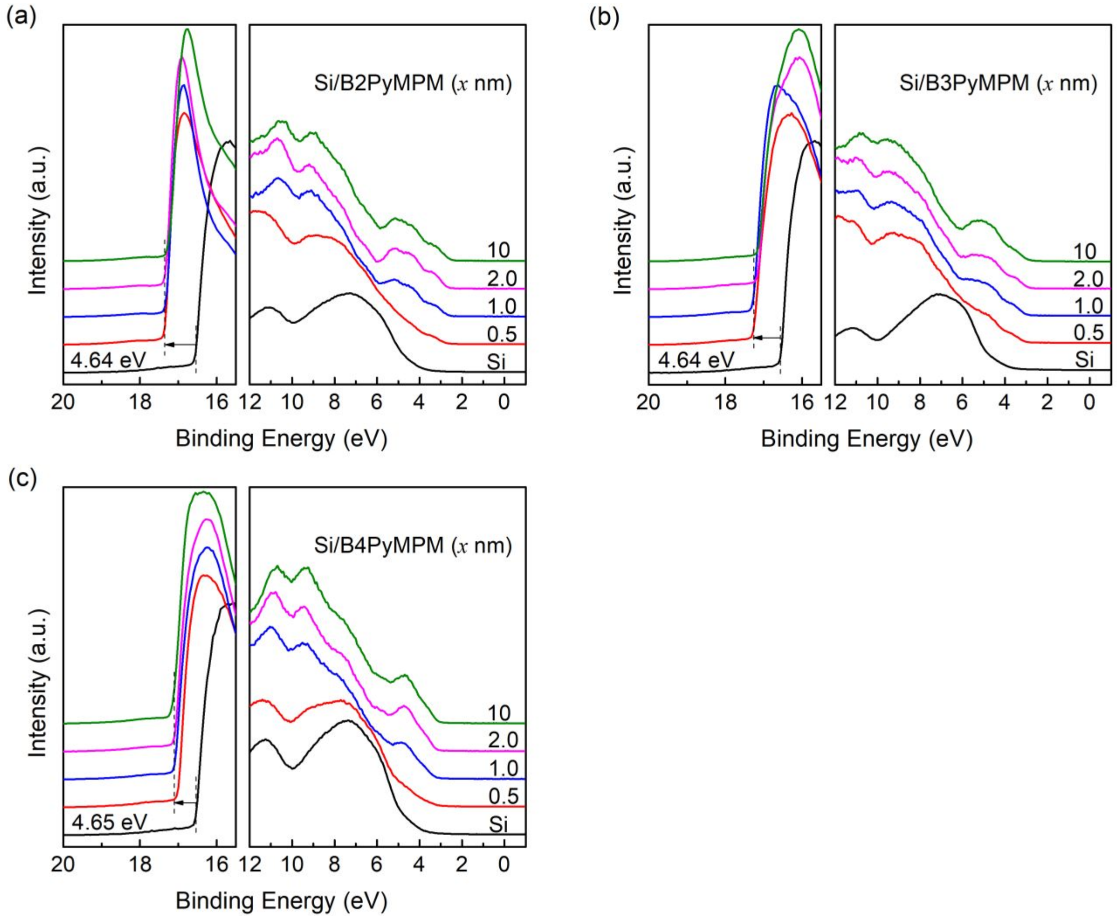

Figure S5. Evolution of UPS spectra with the increase of film thickness: (a) B2PyMPM, (b) B3PyMPM, (c) B4PyMPM. All films are vacuum-deposited on Si/SiO_x and the thickness is calibrated by surface profile measurement.

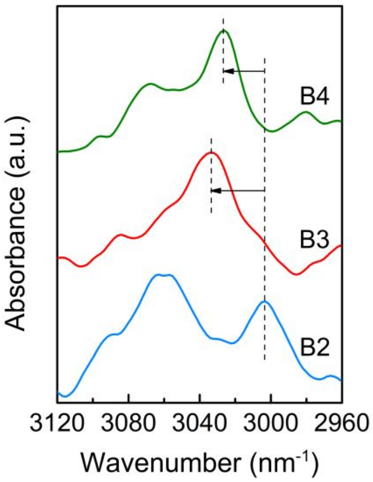

Figure S6. FTIR spectra of the vacuum-deposited films of B2–B4PyMPM. The blue shift of the main bands in B3 and B4PyMPM are marked by arrows.

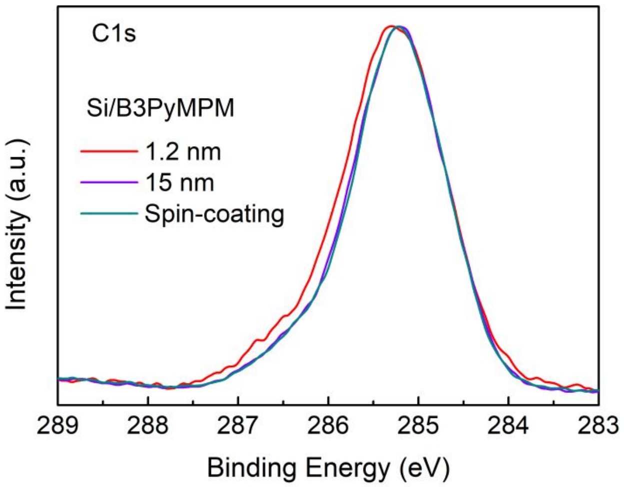

Figure S7. Comparison of C 1s spectra between vacuum-deposited 1.2 nm-thick, 15 nm-thick and spin-coated films.

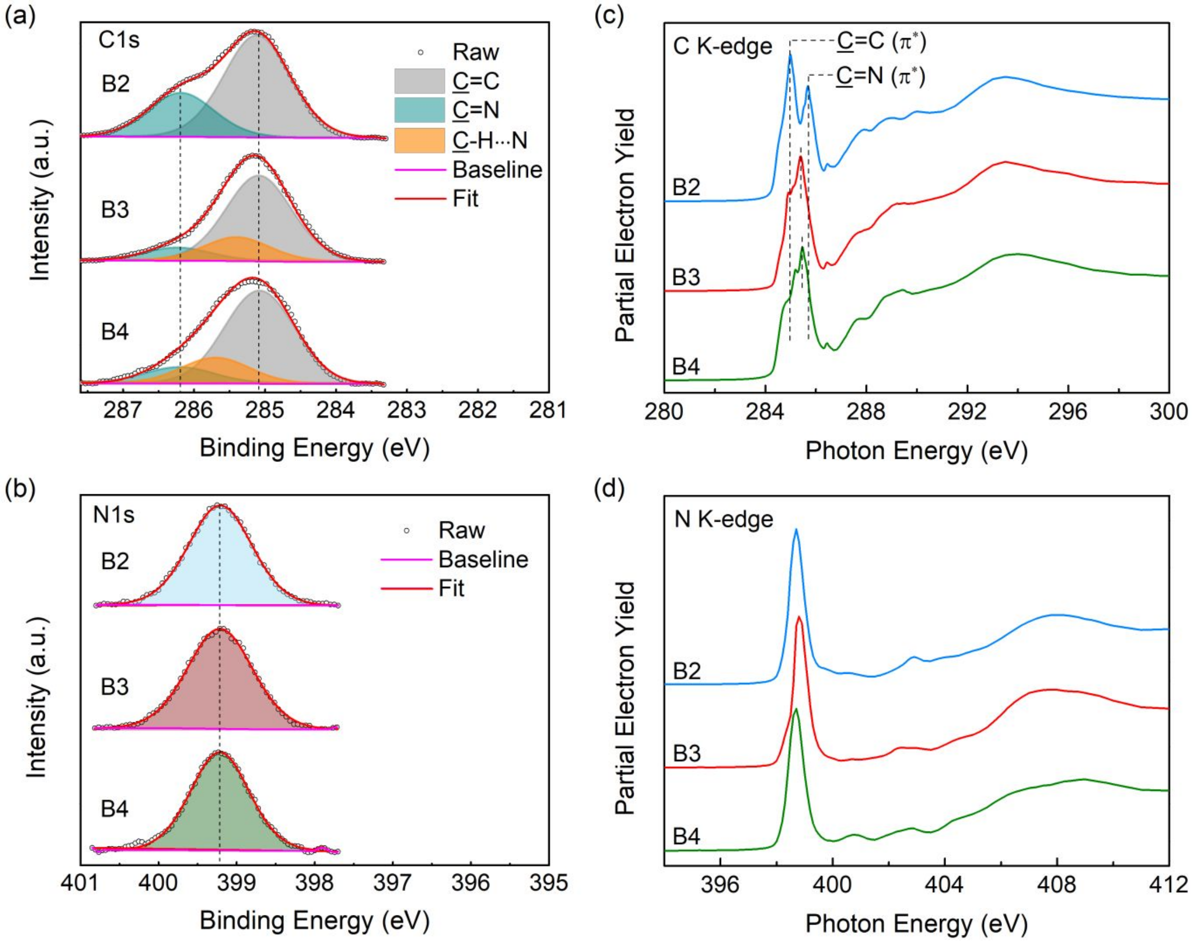

Figure S8. (a) C 1s and (b) N 1s XPS spectra with simple peak fitting measured from the vacuum-deposited films. The spectra of B3 and B4PyMPM are aligned to that of B2PyMPM. (c) C K-edge and (d) N K-edge NEXAFS spectra of B2-B4PyMPM.

Table S1. C 1s peak fitting parameters of the vacuum-deposited films including binding energy (B.E.), area ratio and FWHM.

	States	B.E. (eV)	FWHM (eV)	Ratio (%)
B2PyMPM	C=C	285.11	1.10	69.9
	C=N	286.18	1.10	30.1
B3PyMPM	C=C	285.10	1.10	70.4
	C=N	286.24	1.10	10.6
	C–H···N	285.40	1.08	19.0
B4PyMPM	C=C	285.07	1.16	69.9
	C=N	286.20	1.14	11.9

C–H···N	285.69	1.12	18.2

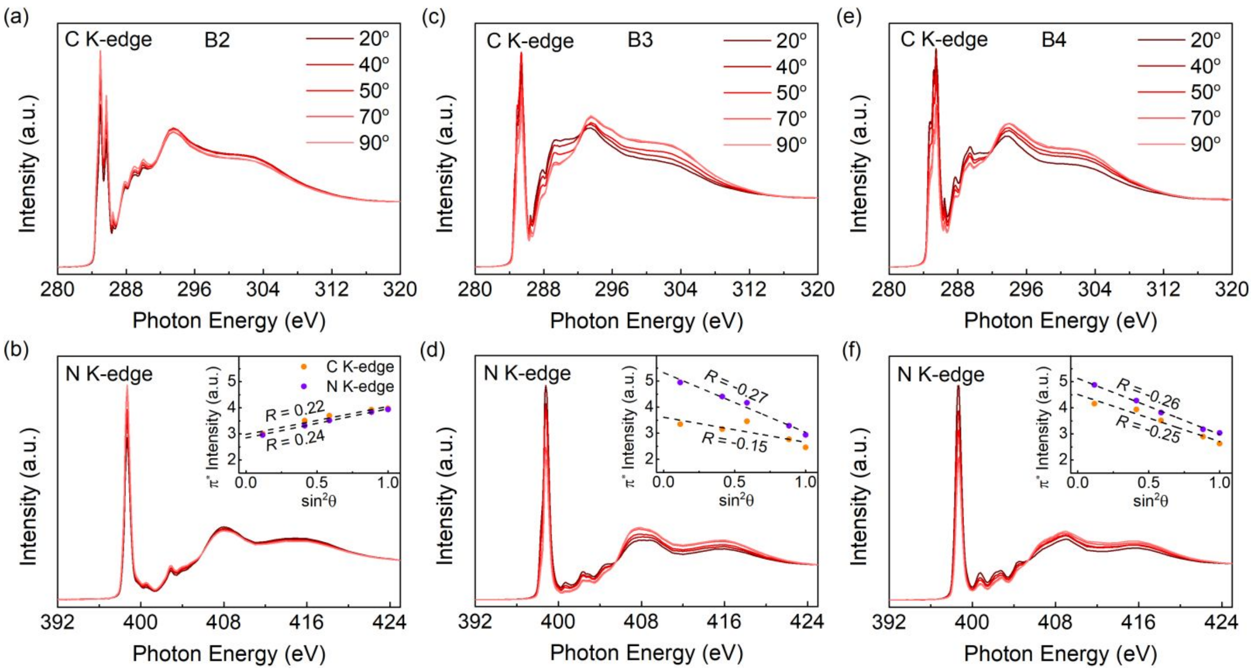

Figure S9. The angle-resolved NEXAFS spectra of carbon (a, c, e) and nitrogen (b, d, f) measured from 1.2 nm-thick vacuum-deposited films at varying incident angles from 20˚ to 90˚. The insets show the dichroic ratio R calculated from the fitting results.

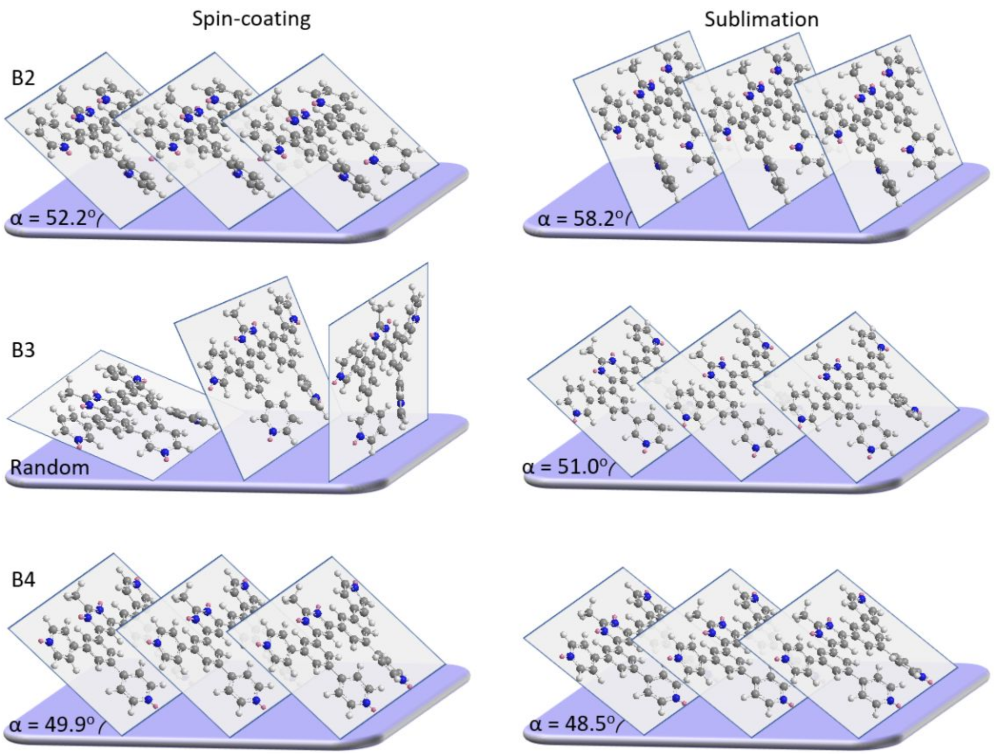

Figure S10. Schematic diagrams of the molecular orientations. The angle-resolved NEXAFS measurement only provides the tilt angle of the molecules, but it cannot determine which edge is in contact with the substrate. Here, we only give one possible geometry. The influence of molecular orientation on the interface dipole depends on the positions of N atoms. Thereinto, the tilt angle has the smallest effect in B3PyMPM, resulting in a similar interface dipole for the films prepared by two methods. For B2PyMPM, a smaller tilt angle benefits for the contact between the lone pair of electrons and substrate, as shown in Figure S11. Therefore, the spin-coated films with smaller tilt angle show larger interface dipole. It is opposite for B4PyMPM, which shows the largest interface dipole when molecules are perpendicular to the substrate. Both spin-coated

and vacuum-deposited B4PyMPM films show similar face-on orientation. Combined with the influence of hydrogen bonds, the interface dipoles formed in B4PyMPM films are always small.

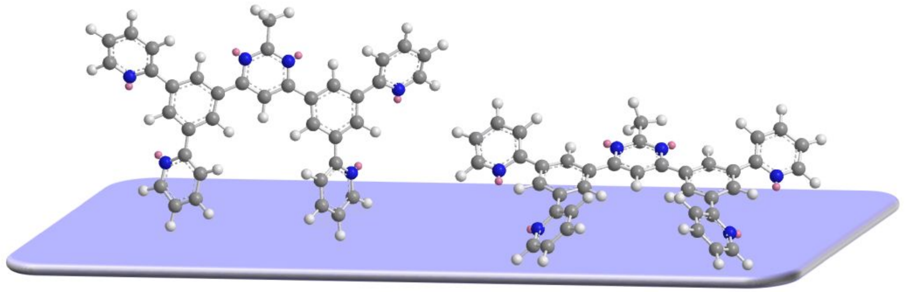

Figure S11. Diagrams of B2PyMPM molecule on the substrate with edge-on (left) and face-on (right) orientation. The nitrogen nuclei are marked in blue and the lone pairs of electrons are marked in pink. The inside nitrogen makes the lone pairs hardly contact the substrate in an edge-on orientation, whereas the non-coplanar conformation enables the lone pairs to move closer to the substrate in a face-on orientation.
